# Chloroplastic Serine Hydroxymethyltransferase From *Medicago truncatula*: A Structural Characterization

**DOI:** 10.3389/fpls.2018.00584

**Published:** 2018-05-11

**Authors:** Milosz Ruszkowski, Bartosz Sekula, Agnieszka Ruszkowska, Zbigniew Dauter

**Affiliations:** ^1^Synchrotron Radiation Research Section of MCL, National Cancer Institute, Argonne, IL, United States; ^2^Department of Chemistry & Biochemistry, University of Notre Dame, Notre Dame, IN, United States

**Keywords:** tetrahydrofolate, pyridoxal 5′-phosphate, one-carbon, serine metabolism, glycine metabolism, glycolate pathway, crystal structure

## Abstract

Serine hydroxymethyltransferase (SHMT, EC 2.1.2.1) is a pyridoxal 5′-phosphate (PLP)-dependent enzyme which catalyzes the reversible serine-to-glycine conversion in either a tetrahydrofolate-dependent or -independent manner. The enzyme is also responsible for the tetrahydrofolate-independent cleavage of other β-hydroxy amino acids. In addition to being an essential player in the serine homeostasis, SHMT action is the main source of activated one-carbon units, which links SHMT activity with the control of cell proliferation. In plants, studies of SHMT enzymes are more complicated than of those of, e.g., bacterial or mammalian origins because plant genomes encode multiple SHMT isozymes that are targeted to different subcellular compartments: cytosol, mitochondria, plastids, and nucleus. Here we report crystal structures of chloroplast-targeted SHMT from *Medicago truncatula* (*Mt*SHMT3). *Mt*SHMT3 is a tetramer in solution, composed of two tight and obligate dimers. Our complexes with PLP internal aldimine, PLP-serine and PLP-glycine external aldimines, and PLP internal aldimine with a free glycine reveal structural details of the *Mt*SHMT3-catalyzed reaction. Capturing the enzyme in different stages along the course of the slow tetrahydrofolate-independent serine-to-glycine conversion allowed to observe a unique conformation of the PLP-serine γ-hydroxyl group, and a concerted movement of two tyrosine residues in the active site.

## Introduction

The metabolic role of L-serine (Ser) reaches far beyond being a building block of proteins. Ser acts in a number of cellular processes, of which particularly interesting is the generation of one-carbon units (Kalhan and Hanson, 2012; Ros et al., 2014), required for the synthesis of vital metabolites, such as thymidylate and methionine. Thus, serine metabolism is related to the control of cell proliferation; in fact, many reports have shown links to cancer development (Amelio et al., 2014a,b; Antonov et al., 2014; Labuschagne et al., 2014). The one-carbon units result mainly from the activity of serine hydroxymethyltransferases (SHMTs, EC 2.1.2.1), which reversibly interconvert Ser and glycine (Gly). Consistently, an increasing amount of evidence has pinpointed SHMTs as pivotal in highly proliferating cells (Girgis et al., 1997; Townsend et al., 2004; Wu et al., 2017).

SHMTs are α-class pyridoxal 5′-phosphate (PLP)-dependent enzymes (Alexander et al., 1994) which transfer hydroxymethyl of Ser to – typically polyglutamylated (polyGlu) – tetrahydrofolate (H_4_PteGlu_n_), producing Gly and 5,10-CH_2_-H_4_PteGlu_n_ (Chen and Schirch, 1973b). The currently proposed mechanism of SHMT activity involves a nucleophilic attack by N5 of H_4_PteGlu_n_ on the Cβ of PLP-Ser external aldimine (PLP-Ser) with displacement of the Cα of Gly (Schirch and Szebenyi, 2005). This nucleophilic displacement mechanism satisfies most of experimental evidence but a retroaldol mechanism, involving PLP-Ser cleavage to formaldehyde which subsequently reacts with H_4_PteGlu_n_, has not been conclusively excluded. The retroaldol mechanism is the route for SHMT-catalyzed cleavage of other β-hydroxy amino acids in H_4_PteGlu_n_-independent reactions. In the context of this work it is also important to note that H_4_PteGlu_n_ is not essential even for the Ser-to-Gly conversion (with the release of formaldehyde), which can proceed in the absence of H_4_PteGlu_n_, albeit at a much slower rate (Chen and Schirch, 1973a). Interestingly, despite over 50 years of SHMT studies, identification of the catalytic base that abstracts the proton in the H_4_PteGlu_n_-independent retroaldol reaction has remained baffling. Although the H_4_PteGlu_n_-independent Ser-to-Gly conversion is of a rather minor importance *in vivo*, the insights in its mechanism deliver important information about the function of this enzyme in general.

Plant genomes encode several SHMT sequences; e.g., seven in *Arabidopsis thaliana* (*At*) (Hanson and Roje, 2001; Zhang et al., 2010). The plant SHMT isoforms have different cellular localization: mitochondrial, cytosolic, chloroplastic, and nuclear (Zhang et al., 2010). Moreover, at least some of SHMT isoforms are controlled by the circadian clock (*At*SHMT1 and *At*SHMT4), which is consistent with their involvement in photorespiration (McClung et al., 2000). Also, the use of H_4_PteGlu_n_ (or 5,10-CH_2_-H_4_PteGlu_n_, depending on the reaction direction) synchronizes activity of SHMT enzymes with the glycine cleavage system (GCS; Douce et al., 2001; Kikuchi et al., 2008). As a result, in photorespiration (Bauwe et al., 2010; Maurino and Peterhansel, 2010), the equilibrium of SHMT-catalyzed reaction is shifted towards the thermodynamically non-favored Ser synthesis due to an increased activity of GCS (high 5,10-CH_2_-H_4_PteGlu_n_/H_4_PteGlu_n_ ratio) in the mitochondrial matrix (Rebeille et al., 1994). The glycolate pathway, where SHMT acts to biosynthesize Ser in plants, is one of the three routes of Ser biosynthesis; others are glycerate and phosphorylated pathways (Ros et al., 2014).

Because of their key roles in one-carbon donation and Ser biosynthesis, SHMT enzymes are recognized as attractive targets for antitumor, antibiotic, and herbicide design (Renwick et al., 1998; Daidone et al., 2011). A myriad of structures of SHMTs – from other domains of life – with inhibitors has been reported (Schwertz et al., 2017a,b). Some of the inhibitors exhibited low-nanomolar IC_50_ against *At*SHMT in the functional assays (the authors did not specify which of the seven isoforms).

Here we report crystal structures of a chloroplastic SHMT enzyme from the model legume plant, *Medicago truncatula* (*Mt*), which similarly to *At* has seven SHMT isoforms. The object of this study from now on will be referred to as *Mt*SHMT3 due to its closest identity to the chloroplastic *At*SHMT3 (74/81% identity/similarity for the entire sequence or 83/90% for the protein lacking the target peptide). *Mt*SHMT3 crystals soaked with selenourea served to solve the structure experimentally by single anomalous dispersion (SAD) phasing. High-resolution diffraction data, collected from crystals in different states, allowed to capture structural snapshots along the course of the enzymatic reaction. Moreover, we provide an updated phylogenetic analysis of plant SHMTs with a special emphasis on the subcellular compartmentalization of SHMT isozymes.

## Materials and Methods

### Cloning, Overexpression, and Purification of *Mt*SHMT3

*Mt*SHMT3 was obtained using a modified protocol recently applied for the production of *M. truncatula* L-histidinol dehydrogenase (Ruszkowski and Dauter, 2017). Briefly, the total RNA was isolated from *M. truncatula* leaves using the RNeasy Plant Mini Kit (Qiagen), and was reverse-transcribed into the complementary DNA (cDNA) with SuperScript II reverse transcriptase (Life Technologies). The chloroplast-targeting peptide was recognized using the TargetP 1.1 server (Nielsen et al., 1997; Emanuelsson et al., 2000), and based on sequence homology with other plant homologs, the construct was designed to yield a peptide N-truncated at F82. The open reading frame coding for *Mt*SHMT3 82-533 fragment was amplified by polymerase chain reaction. The primers used (forward: TACTTCCAATCCAATGCCTTCTTGGACTATGGCTTGAGTGAAGCT, reverse: TTATCCACTTCCAATGTTATTAGACTCCAGGAATAGGATATTGAGTAG) were compatible with the pMCSG68 vector (Midwest Center for Structural Genomics) and the expression plasmid was created by a ligase-independent cloning method (Kim et al., 2011). The protein expressed from pMCSG68 vector contains an N-terminal His_6_-tag, followed by the Tobacco Etch Virus (TEV) protease cleavage site and an Ser–Asn–Ala linker. The correctness of the insert was confirmed by DNA sequencing.

Overexpression was carried out in BL21 Gold *E. coli* cells (Agilent Technologies) in LB media supplemented with 150 μg/mL ampicillin. The bacteria were cultured with shaking at 190 rpm at 37°C until the A_600_ reached 1.0. Afterwards, the cultures were chilled to 18°C, and isopropyl-D-thiogalactopyranoside was added at a final concentration of 0.5 mM to trigger overexpression which continued for 18 h. The cell pellet from the 2 L culture was centrifuged at 3,500 × *g* for 30 min at 4°C and resuspended in 35 mL of binding buffer [50 mM Hepes-NaOH pH 7.5; 500 mM NaCl; 20 mM imidazole; 1 mM tris(2-carboxyethyl)phosphine (TCEP)] and stored at -80°C.

The cells were disrupted by sonication in an ice/water bath using bursts of 4 s and 26 s intervals for a total of 5 min of the probe working time. The lysates were cleared by centrifugation at 25,000 × *g* for 30 min at 4°C. The supernatant was poured into a 50 mL column packed with 3 mL of HisTrap HP resin (GE Healthcare) plugged into vacuum pump-VacMan setup (Promega). The resin-bound His_6_-tagged *Mt*SHMT3 was washed six times with 40 mL of the binding buffer. Then, the protein was eluted with 20 mL of elution buffer (50 mM Hepes-NaOH pH 7.5; 500 mM NaCl; 400 mM imidazole; 1 mM TCEP). The imidazole concentration was lowered to 20 mM by dialysis overnight at 4°C and, simultaneously, the His_6_-tag was cleaved with TEV protease (at final concentration 0.1 mg/mL). The sample was transferred to the second HisTrap column, and the flow-through (containing *Mt*SHMT3) was collected in which the cleaved His_6_-tag and the His_6_-tagged TEV protease had been eliminated. The sample was concentrated to 2.4 mL and applied on a HiLoad Superdex 200 16/60 column (GE Healthcare), equilibrated with a buffer composed of 25 mM Hepes-NaOH pH 7.5, 100 mM KCl, 50 mM NaCl, and 1 mM TCEP. The standard curve for the column was based on thyroglobulin (670 kDa), gamma-globulin (158 kDa), ovalbumin (44 kDa), and myoglobin (17 kDa) from the Gel Filtration Standard (BioRad).

### Crystallization and Diffraction Data Collection

The tetrameric fraction of *Mt*SHMT3 was concentrated using centrifugal concentrators (Millipore) to 31 mg/mL (based on A_280_ with the extinction coefficient of 26,400). The crystals were grown by vapor diffusion method in hanging drops containing 2 μL of each, the protein and reservoir solutions. *Mt*SHMT3 holo/apo crystals grew in 55% Tacsimate pH 7.0 in the reservoir. Cryoprotection was obtained by washing the crystals with 100% solution of Tacsimate pH 7.0. Crystals for SAD phasing data collection were obtained from the same condition but were transferred into a 2 μL drop of 100% Tacsimate pH 7.0 with a single crystal of selenourea (∼0.5 mm × 0.1 mm × 0.1 mm) and soaked for 15 min. The complexes showing the reaction intermediates were obtained from crystals grown in 75 mM MES [2-(*N*-morpholino)ethanesulfonic acid] pH 6.5, 19% polyethylene glycol (PEG) 3350 and 150 mM ammonium acetate. The mature crystals were soaked with 200 mM Ser for 2 h and cryoprotected by the addition of ethylene glycol to a final concentration of 20%. All crystals were flash-frozen in liquid nitrogen and stored for diffraction data collection. Data were collected at 19-ID and 22-ID beamlines at the Advanced Photon Source, Argonne, United States. The diffraction images were processed with *XDS* (Kabsch, 2010). The statistics of the data collection and processing are summarized in **Table 1**.

**Table 1:** Data collection and refinement statistics.

*Mt*SHMT3	holo/apo	Ser-soaked	selenourea-soaked
**Data collection**			
Beamline	APS 22-ID	APS 19-ID	APS 22-ID
Wavelength (Å)	1.0000	0.9792	0.9778
Space group	*P*2_1_2_1_2	*P*2_1_	*C*222_1_
Unit cell parameters			
*a, b, c* (Å)	151.7, 201.6, 64.8	94.1, 103.7, 180.4	64.7, 199.7, 152.4
α, β, γ (°)	90, 90, 90	90, 97.4, 90	90, 90, 90
Resolution (Å)	84–1.74 (1.84–1.74)	46.7–1.91 (2.02–1.91)	100–2.40 (2.46–2.40)
Unique reflections	203236 (32184)	263165 (41826)	74396 (5340)
Multiplicity	4.5 (4.3)	4.7 (4.7)	12.2 (7.3)
Completeness (%)	99.6 (98.5)	98.6 (97.3)	99.8 (97.6)
*R*_meas_^a^(%)	6.3 (106.2)	7.1 (82.6)	11.3 (84.9)
<*I*/σ(*I)*>	16.7 (1.9)	13.3 (1.9)	23.3 (5.9)
CC_1/2_	99.9 (77.8)	99.9 (76.9)	99.9 (95.6)
**Refinement**			
*R*_free_ reflections	1017	1316	1007^b^
No. of subunits per asymmetric unit	4	8	2
No. of atoms (non-H)	15607	29964	7226
protein	14102	27732	6783
ligands	96	200	118
solvent	1409	2032	325
*R*_work_/*R*_free_ (%)	17.5/21.9	19.1/23.6	16.6/20.6
Average B-factor (Å^2^)	33	41	35
rmsd from ideal geometry			
bond lengths (Å)	0.010	0.012	0.014
bond angles (^o^)	1.4	1.5	1.6
Ramachandran statistics (%)			
favored	97	97	96
allowed	3	3	4
outliers	0	0	0
PDB code	6cd0	6cd1	6ccz

Values in parentheses correspond to the highest resolution shell. ^*a*^*R*_*meas*_ = redundancy independent R-factor (Diederichs and Karplus, 1997). ^*b*^After phasing, the model was refined against data extended to 2.14 Å resolution.

### Determination and Refinement of the Crystal Structures

The crystal structure of *Mt*SHMT3 was solved by SAD using the recently developed method of soaking crystals with selenourea (Luo, 2016). For phasing, data from two crystals were merged. The phasing was performed with *SHELXC/D/E* (Sheldrick, 2008) under the *HKL2MAP* interface (Pape and Schneider, 2004). The initial model was built using 2.14 Å data from one of the crystals used for phasing with *Phenix AutoBuild* (Terwilliger et al., 2008), and was placed inside the unit cell with the *ACHESYM* server (Kowiel et al., 2014). *COOT* (Emsley et al., 2010) was used for manual fitting in the electron density maps between rounds of model refinement in *Refmac* (Murshudov et al., 2011) with *TLS* (Winn et al., 2003) groups. The refined model served to solve the other two non-isomorphous structures by molecular replacement with *PHASER* (McCoy et al., 2007). The refinement statistics are listed in **Table 1**.

### Other Software Used

Molecular figures were created with UCSF *Chimera* (Pettersen et al., 2004), which also served for calculations of root-mean-square-deviations (rmsds). Sequence alignment for the calculation of the small phylogenetic tree showing *Mt* and *At* sequences was performed using *ClustalW* (Thompson et al., 2002), under *MEGA7* (Kumar et al., 2016) suite whereas for the large tree *MUSCLE* (Edgar, 2004) was employed to align 711 protein sequences. The surface conservation was calculated using *ConSurf* (Ashkenazy et al., 2016) based on the alignment file (this work). Surface electrostatic potential was calculated using *PDB2PQR* and *APBS* servers (Baker et al., 2001; Dolinsky et al., 2004). Identities/similarities were calculated in *BLAST* (Altschul et al., 1990). Signal peptides were predicted with *WoLF PSORT* (Horton et al., 2007), *SeqNLS* (Lin and Hu, 2013), and *TargetP* (Emanuelsson et al., 2000) webservers.

## Results and Discussion

### Phylogenetic Analysis of Plant SHMTs

**FIGURE 1:**
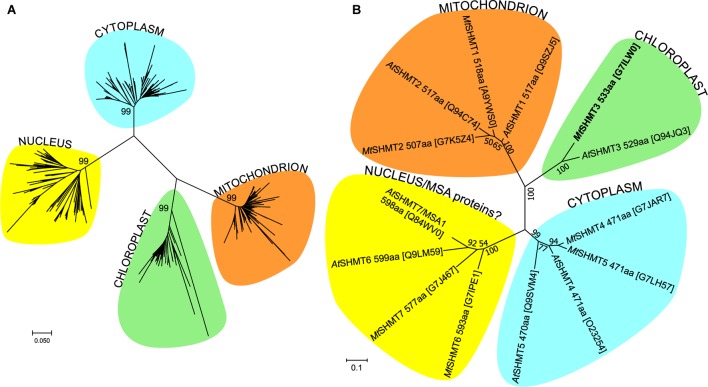
Phylogenetic analysis of SHMT protein sequences. Panel **(A)** shows the evolutionary history within *Magnoliophyta*, inferred using the Neighbor-Joining method (Saitou and Nei, 1987). The optimal tree with the sum of branch length = 14.6 is shown. The analysis involved 711 amino acid sequences. Panel **(B)** shows the tree with the highest log likelihood (–5970) of SHMT sequences from *Arabidopsis thaliana* and *Mt*. The Maximum Likelihood method based on the JTT matrix-based model (Jones et al., 1992) was used to analyze the 14 sequences (446 positions). The trees are drawn to scale in the number of amino acid substitutions per site. UniProt accession numbers are given in square brackets in **(B)**. Evolutionary analyses were conducted in MEGA7 (Kumar et al., 2016).

**FIGURE 2:**
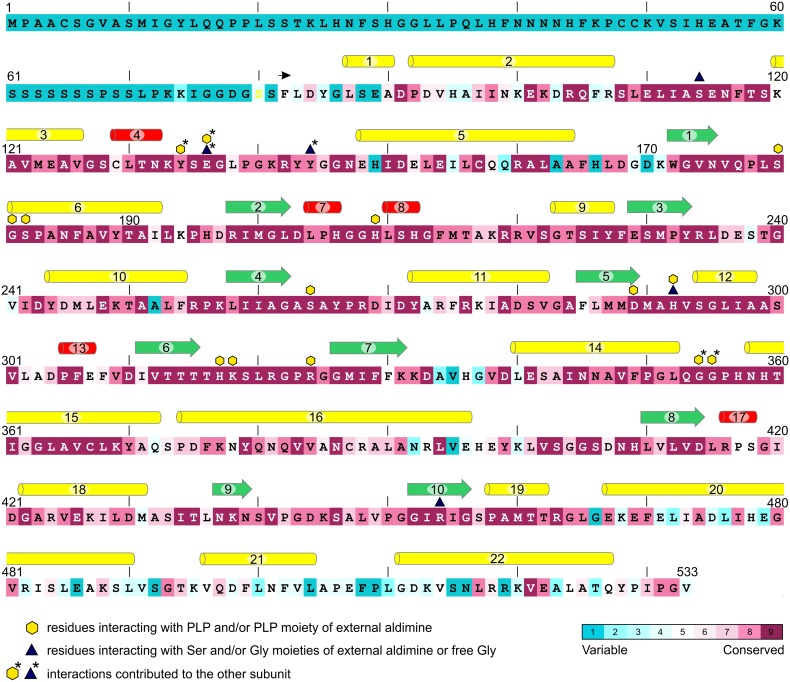
Conservation of *Mt*SHMT3 sequence in comparison to SHMTs from flowering plants. In total, 711 sequences were aligned and analyzed; the coloring scheme is shown in the lower-right corner. The variable N-terminal fragment contains the chloroplast-targeting peptide. The construct used in this study starts from F82 (black arrow). Secondary structure elements are visualized as: yellow pipes, α-helices; red pipes, 3_10_ helices; green arrows, β-strands. Residues interacting with PLP, Ser or Gly moieties of external aldimines and/or free Gly are marked according to the legend.

The flowering plant SHMT sequences group into four clades (**Figure 1**), each containing proteins of different subcellular localization. More precisely, within the respective branches 80% cytosolic, 81% mitochondrial, 62% chloroplastic, and 62% nuclear proteins were recognized by the used prediction software. Due to the presence or absence of organelle-targeting peptides, lengths of the protein sequences between the branches vary significantly, as the mode values are 471, 517, 529, and 565 amino acid residues for the cytosolic, mitochondrial, chloroplastic, and nuclear isoforms, respectively. It is also very interesting to note that among the analyzed SHMTs that contain a nuclear localization signal, 38% are predicted to also hold an N-terminal chloroplast-targeting peptide. In summary, SHMTs from different species but of the same subcellular compartment are more similar than isozymes from the same species but of a different localization. Moreover, all SHMT sequences from the flowering plants show significant homology, except for their N- and C- terminal regions (**Figure 2**) that can contain organelle-targeting peptides. Our updated analysis of 711 sequences from *Magnoliophyta* (flowering plants) division, annotated as SHMTs (Family IPR001085) in the InterPro database (Finn et al., 2017) is in agreement with the previous report from Zhang et al. (2010), which was limited to 49 sequences.

Of the seven *A. thaliana* SHMT isoforms (*At*SHMT1-7; **Figure 1B**), *At*SHMT1-2 are targeted to mitochondria (McClung et al., 2000; Heazlewood et al., 2004; Huang et al., 2015), *At*SHMT3 exists in plastids (Zhang et al., 2010), *At*SHMT4-5 contain no signal peptide thus localize in the cytoplasm, whereas *At*SHMT6-7 hold nuclear-targeting signals (Zhang et al., 2010). However, the nuclear *At*SHMT7 has been recently shown to actually lack SHMT activity *in vitro* and – to better reflect its role – was renamed to “more sulfur accumulation1” (MSA1) protein (Huang et al., 2016). The exact molecular function of *At*MSA1 is unknown but the authors suggested it may regulate the nuclear sulfur homeostasis through a control of *S*-adenosylmethionine levels. Moreover, *At*SHMT7 is very similar to *At*SHMT6 but there is no experimental evidence whether or not *At*SHMT6 possesses SHMT activity. On the other hand, since SHMT activity was reported in pea nuclei (Neuburger et al., 1996) at least one actual SHMT isoform is expected to exist in the plant nucleus.

In *M. truncatula*, after removing database sequences that are redundant or incomplete or could not be mapped to any locus in the genome, there are seven SHMT isoforms (numbering corresponds to their closest *A. thaliana* homologs): two mitochondrial (*Mt*SHMT1-2), one chloroplastic (*Mt*SHMT3), two cytosolic (*Mt*SHMT4–5), and two nuclear (*Mt*SHMT6–7) (**Figure 1B** and Supplementary Figure S1). Importantly, neither *Mt*SHMT6 nor *Mt*SHMT7 were tested for SHMT activity to assess if they function as *At*MSA1.

### The Tetrameric Structure of *Mt*SHMT3 Resembles Mammalian Homologs

The crystal structure of *Mt*SHMT3 (residues 82–533), which is the first structure of a plant SHMT, was solved using the recently developed phasing method that utilizes selenourea soaking (Luo, 2016). Based on the anomalous difference maps, at least twenty selenourea molecules were bound to the protein, often *via* extensive networks of hydrogen bonds (Supplementary Figures S2A–D).

**FIGURE 3:**
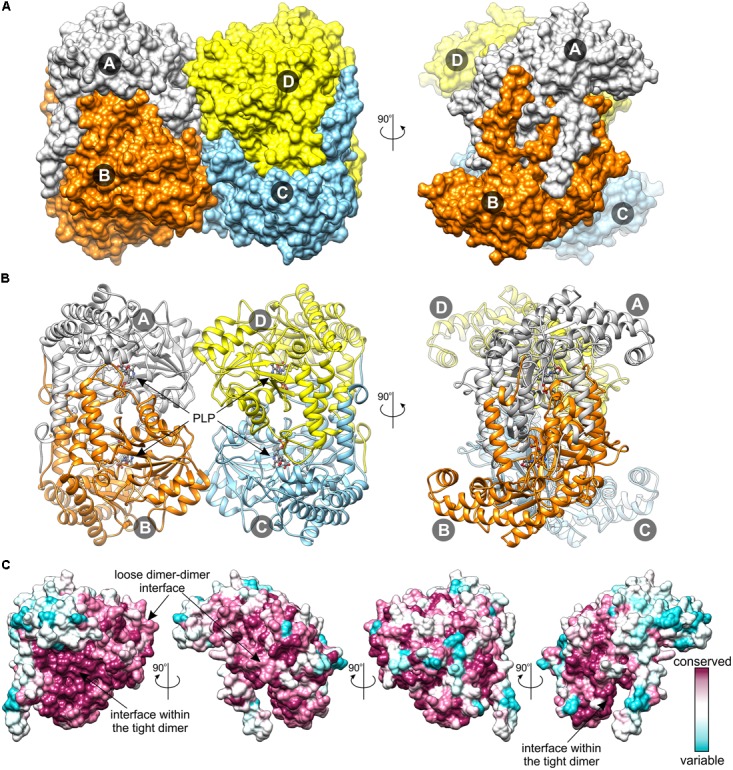
Structure of *Mt*SHMT3 tetramer. Surface representation **(A)** shows vast intersubunit interfaces within the tight, vertically arranged dimers and much smaller contacts between the two dimers. In panel **(B)**, the secondary structure elements, the location of PLP, indicated by the black arrow, and PLP-binding K318 (both in ball-and-stick) are shown. Panel **(C)** depicts sequence conservation on the surface of *Mt*SHMT3 subunit; the first orientation is as of the A subunit in panel A.

The reported herein crystal structures of *Mt*SHMT3 arose from crystals that were not isomorphous. Nonetheless, the results presented further in the text were cross-validated against subunits showing the same states to mitigate a bias from different packing and/or crystallization conditions on the protein conformation. Superposition of all protein chains from the holo/apo and Ser-soaked structures is shown in Supplementary Figure S3. According to the PISA analysis (Krissinel, 2015), in each case the subunits of *Mt*SHMT3 form stable homotetramers (**Figure 3**). The apparent molecular weight observed in size exclusion chromatography (∼150 kDa, Supplementary Figure S4) is less than a theoretical molecular mass of the tetramer (∼197 kDa). However, this can be attributed to a non-globular shape of the protein (see below), which then penetrates through smaller pores of the resin, and therefore has a retained elution from the column. The tetrameric quaternary assemblies have been reported for mammalian SHMTs: human (Renwick et al., 1998), rabbit (Scarsdale et al., 1999), and mouse (Szebenyi et al., 2000). However, in the human mitochondrial SHMT2 (hmSHMT2), PLP-binding appears to trigger the dimer-to-tetramer transition (Giardina et al., 2015). Moreover, at least two examples from lower eukaryotes, SHMTs of *Plasmodium falciparum* (Chitnumsub et al., 2014a) and *Plasmodium vivax* (Chitnumsub et al., 2014b) form dimers. Prokaryotic SHMTs are generally dimers (Scarsdale et al., 2000; Angelaccio et al., 2014); however, e.g., the enzyme from *Bacillus stearothermophilus* was shown to form both dimers and tetramers (Jala et al., 2002).

Similar to the mammalian homologs, the tetramer of *Mt*SHMT3 (222 symmetry) is formed by two tight dimers (subunits A+B and C+D in **Figure 3**). The interface between the *Mt*SHMT3 subunits that form the obligate tight dimers (necessary to form a complete active site, see below) is three-fold larger (∼4500 Å^2^) than between the dimers in the tetramer (∼1500 Å^2^). It is also interesting to note that the residues at the interface within the tight dimer are more conserved than those at the inter-dimeric face (**Figure 3C**). It is thus possible that among the analyzed sequences of plant SHMTs there may exist isoforms that do not form tetramers but only dimers – as the prokaryotic enzymes do.

**FIGURE 4:**
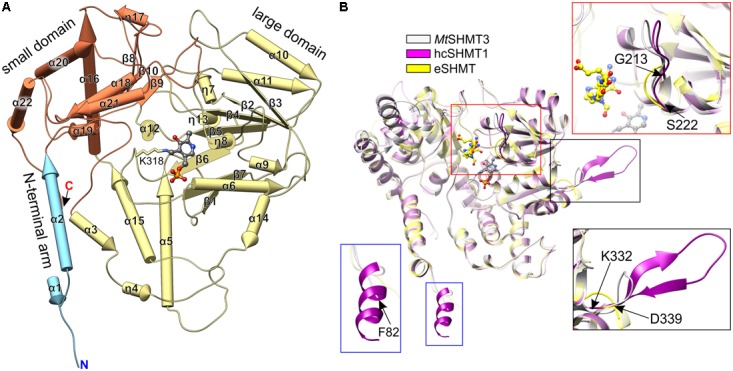
Structure of *Mt*SHMT3 subunit. Secondary structure elements (pipes-and-planks model) are shown in panel **(A)**. N-terminal arm, the large and small domains are light blue, pale yellow, and coral, respectively. Panel **(B)** presents a comparison of *Mt*SHMT3 (chain A, light gray) with human cytosolic hcSHMT1 (PDB ID: 1bj4, magenta; Renwick et al., 1998) and *E. coli* eSHMT [1dfo, chain C, yellow (Scarsdale et al., 2000)]. Similar elements are semitransparent, PLP (from holo-*Mt*SHMT3) and 5-formyl-H_4_PteGlu_1_ (from 1dfo complex) are shown as gray and yellow balls-and-sticks, respectively.

*Mt*SHMT3 is a member of the α-class of PLP enzymes (Alexander et al., 1994), and its overall fold is typical for this family (**Figure 4**). A subunit of *Mt*SHMT3 can be subdivided into three regions: N-terminal arm, large domain, and small domain (**Figure 4A**), consistently with other SHMTs (Scarsdale et al., 2000). The N-terminal arm (residues 82-107), contains helices α1 and α2, and interacts with the other subunit within the tight dimer. In fact, mutations within this region in sheep liver cytosolic SHMT (scSHMT) were shown to destabilize the protein (Jagath et al., 1997). The large domain (residues 120–373) is where the PLP prosthetic group binds at K318. The large domain forms an αβα sandwich of seven-stranded mixed β-sheet (β1↑-β7↓-β6↑-β5↑-β4↑-β2↑-β3↑) shielded by helices α6, η8, α9, and α14 from one side, and helices α5, α10, α11, α12, η13, and α15 from the other. The peptide bond between F349 and P350 is in *cis*-conformation. The small domain (residues 108–119 and 374–533) folds into an αβ sandwich. One face of its antiparallel β-sheet (β8-β10-β9) interacts with the large domain, whereas the other is sheltered by helices α16-α22. Notably, the four Cys residues of *Mt*SHMT3 are distant from each other thus are not involved in a formation of either intra- or inter-subunit disulfide bridges, unlike the case of *Pf*SHMT, whose C125–C364 are controlled by the redox status (Chitnumsub et al., 2014a).

Search among the Protein Data Bank (PDB) (Berman et al., 2000) with the use of Dali server (Holm and Rosenstrom, 2010) revealed that *Mt*SHMT3 structure is most similar to the human cytosolic SHMT1 (hcSHMT1; rmsd = 1.0 Å, *Z* = 62.1, 60% identity; PDB ID: 1bj4, Renwick et al., 1998; **Figure 4B**). The tetrameric architecture and most of the secondary structure elements of *Mt*SHMT3 and human enzyme are the same. However, the most pronounced difference is a presence of an insert in hcSHMT1 (residues K271-N287) which contains a β-hairpin (**Figure 4B**, black frame) of unknown function. The corresponding fragment of *Mt*SHMT3, the loop between β7 and α14 (residues K332-D339), is significantly shorter and with no β-strand conformation. Importantly, this fragment is rather variable in plants (**Figure 2**), and in *Mt*SHMT3 it is actually the shortest among all *M. truncatula* SHMTs (Supplementary Figure S1). This may indicate a specific function that is related to the subcellular localization. Another difference is a presence of an additional helical fragment at the N-terminus of hcSHMT1 which our *Mt*SHMT3 structures lack (**Figure 4B**, blue frame); however, that might be attributed to the design of the crystallized construct.

Structural superposition with a prokaryotic example, *E. coli* SHMT [eSHMT, PDB ID: 1dfo (Scarsdale et al., 2000)] revealed that the protein chains overlap well (**Figure 4B**; rmsd = 1.5 Å, *Z* = 56.2, identity 47%). The most outstanding difference is within the region that maps to the binding site of polyGlu-tail of H_4_PteGlu_n_ (Fu et al., 2003). The loop region 213–222 in *Mt*SHMT3 (**Figure 4B**, red frame) is longer than the corresponding fragment of eSHMT (130–134), and more similar to hcSHMT1 (152–161). Consistently, in *E. coli*, folate derivatives usually contain three Glu residues (Bermingham and Derrick, 2002), in contrast to longer polyGlu-tails of eukaryotes: e.g., plastid folates are typically H_4_PteGlu_4-6_ species (Orsomando et al., 2005). Also, eSHMT is much less sensitive for extension of the H_4_PteGlu_n_ polyGlu chain than *At*SHMT3 or the mammalian examples (Matthews et al., 1982; Stover and Schirch, 1991; Zhang et al., 2010).

### Formation of the PLP Binding Site Involves a Local Disorder-to-Order Rearrangement

**FIGURE 5:**
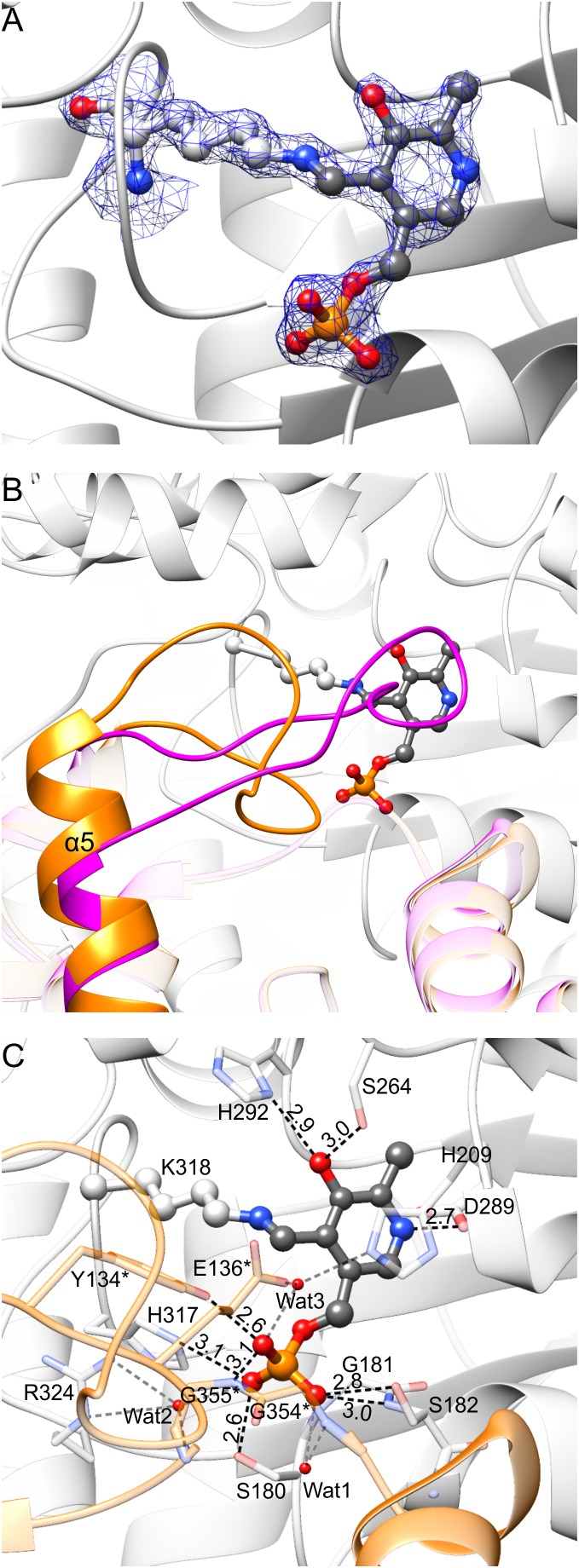
PLP binding by *Mt*SHMT3. In panel **(A)**, blue mesh represents the 2*F_o_–F_c_* map at 1.5 σ level around atoms of the internal aldimine; the corresponding omit *F_o_–F_c_* map is presented in Supplementary Figure S2E. Panel **(B)** shows rearrangement associated with the PLP binding. In the apo-state of subunit A, fragment of subunit B has the conformation shown in magenta; when PLP is bound to subunit A, the fragment of subunit B is reorganized (orange). A detailed PLP binding mode is shown in panel **(C)**; asterisks indicate residues from subunit B. Protein residues are semitransparent. Hydrogen bonds mediated by water molecules (red balls) are gray.

The holo/apo structure of *Mt*SHMT3, with one tetramer in the crystallographic asymmetric unit, shows two states, with and without the PLP prosthetic group. More precisely, two protein chains (A and B) contain the PLP bound as a Schiff base internal aldimine to K318 (holo-state, **Figure 5** and Supplementary Figure S2E) with full occupancy. In one chain there is no cofactor bound (chain C, apo-state), whereas in the chain D, PLP is present at a partial occupancy. Notably, no additional PLP was added during the protein expression or purification, thus the prosthetic group originates solely from the culture.

Comparison of *Mt*SHMT3 subunits in apo- and holo-states revealed that a fragment of the protein undergoes significant rearrangements upon PLP binding (**Figure 5B**). Binding of PLP in one *Mt*SHMT3 subunit is accompanied by conformational changes within residues 133–151 of the other subunit of the tight dimer. For instance, if subunit A is in the apo- state, this entire fragment of its dimer-mate (subunit B) is a loop, contains a *cis*-peptide L138-P139, and the helix α5 starts from E152 (**Figure 5B**, magenta). With PLP bound in subunit A, the peptide bond L138-P139 in subunit B is in *trans*-conformation, and the helix α5 gains an additional twist to start from E148 – leaving only residues 133–147 in the loop region (**Figure 5B**, orange). Two of the loop residues, Y134^∗^ and E136^∗^ (an asterisk indicates a residue from the other subunit of the tight, obligate dimer) hydrogen-bond PLP in the dimer mate (**Figure 5C**, see below), which is likely the reason that drives such disorder-to-order transition.

An extensive network of non-covalent interactions secures PLP internal aldimine in the large domain of *Mt*SHMT3 (**Figure 5C**). The pyridine ring is stacked with H209. The O3 of PLP forms hydrogen bonds with Nδ of H292 and Oγ of S264. The N1 (protonated) is H-bonded to Oδ of D289. The phosphate group forms direct hydrogen bonds with Oγ of S180, N𝜀 of H317, backbone N and Oγ of S182, Oη of Y134^∗^, and backbone N of G355^∗^. Three water molecules mediate additional hydrogen bonds: Wat1 with the backbone N of G354^∗^; Wat2 with carbonyl O of G355^∗^ and guanidine moiety of R324; and Wat3 with N𝜀 of H209 and O𝜀 of E136^∗^. The negative charge of phosphate is also stabilized by a positive dipole moment at the N-terminus of α6 helix.

### Complex With PLP-Ser External Aldimine: The Unusual Conformation of PLP-Ser γ-Hydroxyl Group

Soaking with Ser the *Mt*SHMT3 crystal that grew in the presence of PEG and 150 mM ammonium acetate allowed to capture three snapshots along the course of reaction, killing the metaphorical three birds (intermediates) with one stone (crystal) (Supplementary Figures S2F–H). The three different stages, within the asymmetric unit containing two tetramers are: PLP-Ser external aldimine (chains A and D), PLP-Gly external aldimine (chains B and F), and PLP internal aldimine with free Gly (chains E and H). PLP was bound at a partial occupancy or absent altogether in the chains G and C, respectively. Importantly, the reaction proceeded in the crystal despite H_4_PteGlu_n_ was not present in the crystallization milieu. In fact, it is very likely that the absence of H_4_PteGlu_n_ allowed to apprehend the reaction snapshots because in the cosubstrate presence the reaction proceeds too rapidly. Our structures corroborate that, at a slow rate, the Ser-to-Gly conversion occurs in the absence of H_4_PteGlu_n_ with the release of free formaldehyde (Chen and Schirch, 1973a).

**FIGURE 6:**
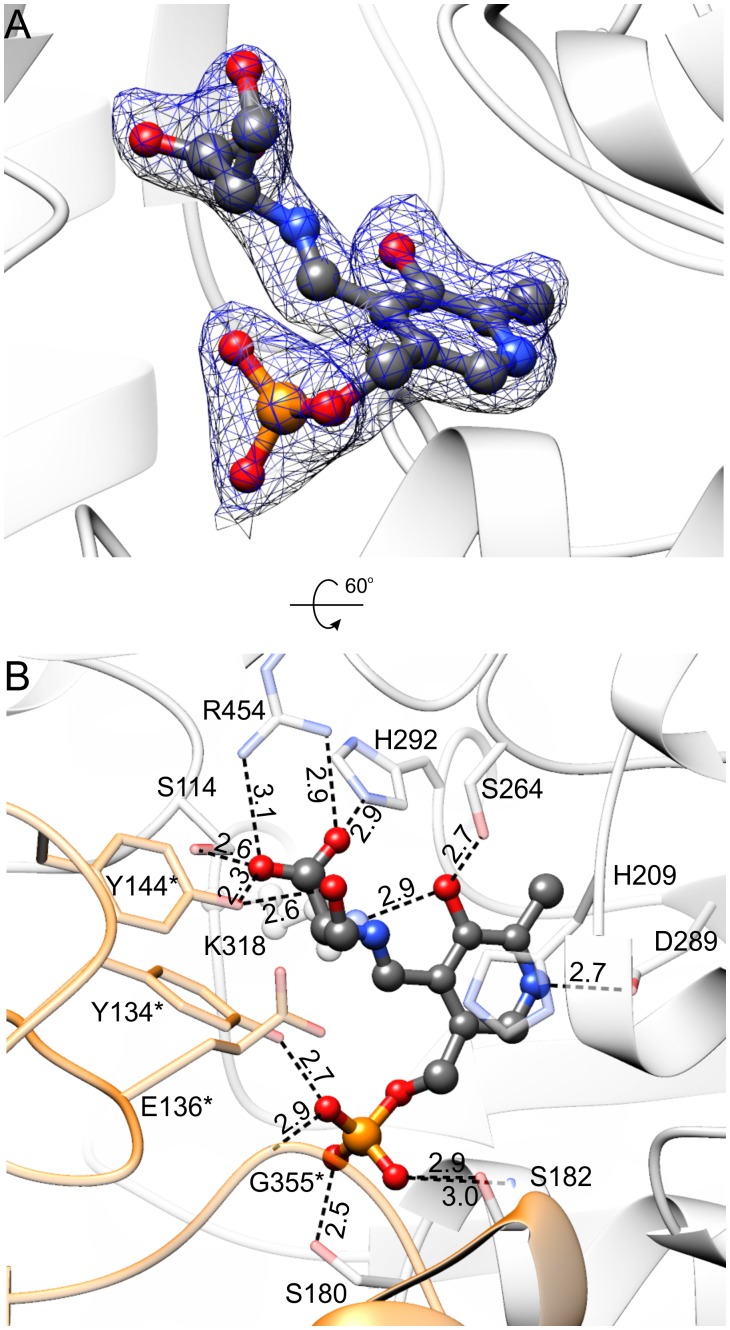
*Mt*SHMT3 in complex with PLP-Ser external aldimine. Electron density maps are shown in panel **(A)**; blue mesh represents 2*F_o_–F_c_* map contoured at 1 σ; the corresponding omit *F_o_–F_c_* map is presented in Supplementary Figure S2F. In panel **(B)**, the hydrogen bonding network is presented; interactions mediated by water molecules are omitted and protein residues are semitransparent for clarity. Subunit A is in light gray, whereas subunit B is orange.

The presence of Ser leads to the formation of Ser-PLP external aldimine (**Figure 6** and Supplementary Figure S2F), which changes the conformation of PLP moiety. The interactions of the phosphate group and the hydrogen bond between protonated N1 atom of pyridine ring with D289 are preserved. However, creation of the covalent bond between PLP and Ser forces the rotation of the plane of the PLP ring ∼20° outwards from Nζ of K318. As a result, O3 no longer H-bonds the side-chain of H292 but interacts with Nζ of K318. The side-chain of H292 is actually flipped to interact with the carboxyl group of the Ser moiety, salt-bridged in turn to R454, and H-bonded to Oγ of S114 and Oη of Y144^∗^. Overall, the environment of R454 guanidinium group is a good placeholder for a carboxylic group, as it binds an acetate anion in the holo-*Mt*SHMT3 structure. The γ-hydroxyl group of the Ser moiety forms a single hydrogen bond with the hydroxyl of Y144^∗^.

It is very intriguing to see that, to our best knowledge, the conformation of the γ-hydroxyl group of PLP-Ser external aldimine is unique in comparison with nearly all other SHMT complexes in the PDB (PDB IDs: 1kkp, 1yjy, 2via, 2vmp, 2vmt, 2vmw, 2w7f, 2w7k, 4ot8), except for the alternative conformation (30% occupancy) in the E53Q mutant of *Geobacillus stearothermophilus* SHMT (bsSHMT, PDB ID: 2vgu Rajaram et al., 2007). In our structure, the γ-hydroxyl is synperiplanar to the PLP-Ser carboxyl carbon and H-bonded to Y144^∗^, whereas in previously reported structures it interacted with residues that correspond to E136^∗^ of *Mt*SHMT3. Role of this conserved glutamate was studied in bsSHMT (E53) and rabbit liver cytosolic SHMT (rcSHMT, residue E75), and it was concluded that it does not participate in the H_4_PteGlu_n_-independent cleavage of L-*allo*-threonine but takes part in the H_4_PteGlu_n_-dependent cleavage of Ser (Szebenyi et al., 2004; Rajaram et al., 2007). Moreover, the very slow formation of formaldehyde (*k*_cat_ of 4.7 × 10^-5^ s^-1^) with wild-type rcSHMT and Ser in the absence of H_4_PteGlu_n_ was actually accelerated by E75L and E75Q mutants (Szebenyi et al., 2004).

Despite years of research, catalytic base that abstracts the hydroxyl proton in the H_4_PteGlu_n_-independent reaction has remained elusive, and the structure of *Mt*SHMT3 with Ser-PLP external aldimine enables a possible explanation for the retroaldol mechanism of Ser cleavage. It is clear that the thermodynamically non-favored synperiplanar conformation of γ-hydroxyl is imposed by the active site architecture of *Mt*SHMT3. In this view, Oη of Y144^∗^, activated by the PLP-Ser carboxyl, might act as the base abstracting the γ-hydroxyl proton from the PLP-Ser external aldimine. This somewhat autocatalytic cleavage could explain why site-directed mutagenesis failed to provide clear answers about the nature of the base. It would also be a simpler interpretation than the mechanism proposed by Bhavani et al. (2008) for L-*allo*-threonine cleavage by bsSHMT (corresponding residue Y61), whereby the Cα proton is abstracted first, followed by an internal rearrangement of the γ-hydroxyl proton to Cα, and cleavage of the Cα-Cβ bond. The role of Tyr residues equivalent to *Mt*SHMT3 Y144^∗^ is very intriguing, as e.g., Y65 of eSHMT was concluded to take part in closed-to-open switching of the active site of the enzyme (Contestabile et al., 2000). Contrastingly, in another study on scSHMT by Rao et al. (2000), the corresponding Y82 was defined to stabilize the quinonoid intermediate. At this point, proton abstraction by carboxyl-activated Y144^∗^ in *Mt*SHMT3, which apparently orients the γ-hydroxyl of PLP-Ser differently than other SHMTs, is merely a possibility. Nevertheless, since we observed reaction intermediates and products (see below), which indicate that the crystals of *Mt*SHMT3 had preserved enzymatic properties, it is likely that the actual snapshots are apprehended in our structures. It is also possible that Y144^∗^ may act as a base only in the H_4_PteGlu_n_-independent cleavage of Ser and thus may have a negligible physiological relevance.

### Charge Distribution on the *Mt*SHMT3 Surface Is Suited to Accept a Polyglutamylated Cosubstrate

**FIGURE 7:**
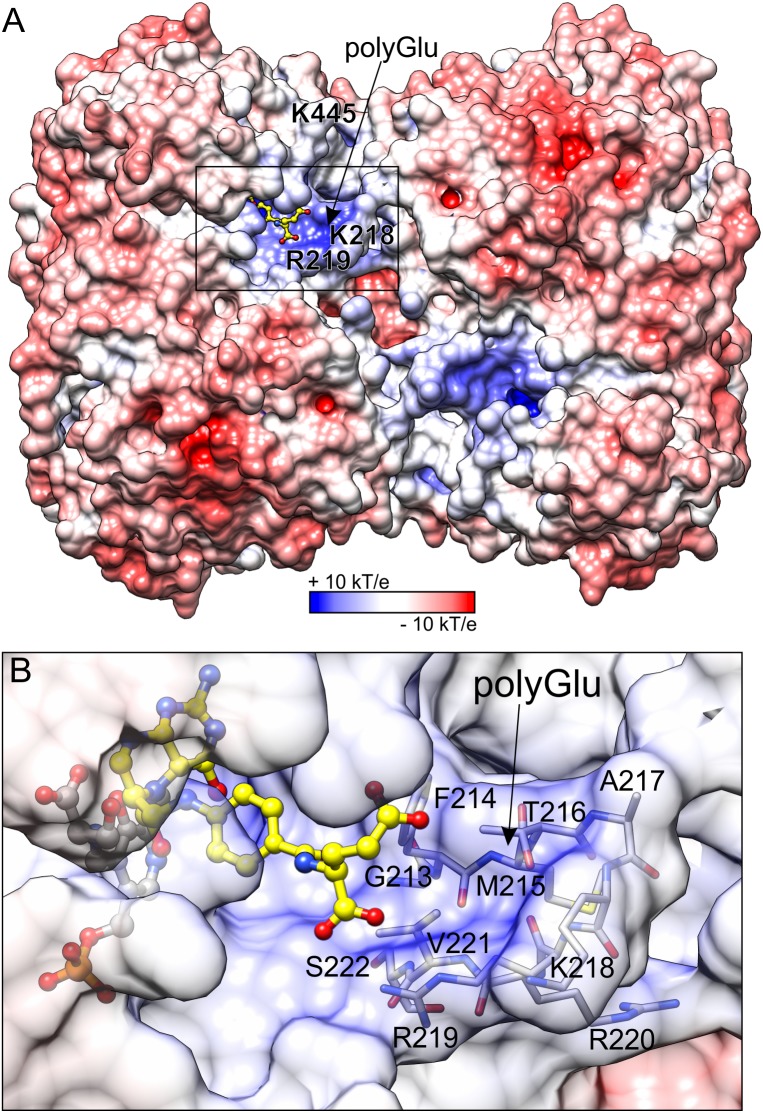
Electrostatic potential distribution mapped on the surface of *Mt*SHMT3. Panel **(A)** shows the entire tetramer, whereas a close-up view of the fragment marked by the black rectangle is presented in panel **(B)**. H_4_PteGlu_1_, superposed from mcSHMT complex (PDB ID: 1eji; Szebenyi et al., 2000), is shown in a yellow ball-and-stick model. The predicted site for polyGlu tail of H_4_PteGlu_n_ is indicated by arrow. The protein surface in panel **(B)** is semitransparent to show residues that form the putative polyGlu binding site.

Distribution of the electrostatic potential on the *Mt*SHMT3 tetramer shows that the channels that lead to the active sites are positively charged as opposed to the rest of the protein surface (**Figure 7**). The entrance to the channel is guarded by two gate loops 213–222 and 440–451; the gate loop 213–222, containing _218_KRR_220_ motif, in particular contributes to the positive charge. It is very interesting to note that neither K218 nor K445 (from the loop 440–451) are strictly conserved (**Figure 2**), and among *M. truncatula* SHMTs are present only in the chloroplast-localized *Mt*SHMT3 (Supplementary Figure S1). The _218_KRR_220_ motif is, however, conserved in *At*SHMT3, which was shown to have *K*_m_ decreasing from ∼218 μM for H_4_PteGlu_1_ to ∼0.64 μM for H_4_PteGlu_5_ (*k*_cat_ decreased from 15.8 to 3.5 s^-1^; Zhang et al., 2010). Consistently, plastid folates are usually H_4_PteGlu_4-6_ species (Orsomando et al., 2005).

Because we were not able to obtain a complex with H_4_PteGlu_n_, we superposed our structure with mouse cytosolic SHMT (mcSHMT) in complex with H_4_PteGlu_1_ (PDB ID: 1eji, chain B, rmsd = 0.76 Å; Szebenyi et al., 2000; **Figure 7**). Based on this composite figure, *Mt*SHMT3 should be able to accommodate H_4_PteGlu_n_ with an extended polyGlu-tail (**Figure 7B**). Unfortunately, we cannot model the active site lineup during the reaction assisted by H_4_PteGlu_n_, but one must keep in mind that it is very likely that the PLP-Ser γ-hydroxyl group may be antiperiplanar or anticlinal to the carboxyl carbon, as has been shown in other SHMTs.

### Structural Changes Associated With Gly Release and the Conformational Switch of Y143^∗^-Y144^∗^

**FIGURE 8:**
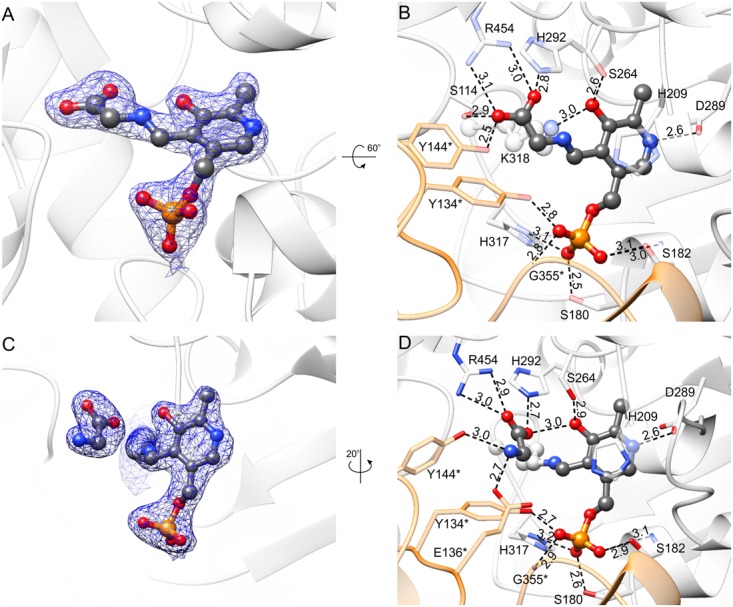
Complexes with PLP-Gly external aldimine **(A,B)** and K318-PLP internal aldimine with free Gly **(C,D)**. 2*F_o_–F_c_* electron density maps (at 1 σ) are shown in panels **(A,C)**; the corresponding omit *F_o_–F_c_* maps are presented in Supplementary Figures S2G,H. Panels **(B,D)** show hydrogen bonding network in the active site; hydrogen bonds mediated by water molecules are omitted and protein residues are semitransparent for clarity.

PLP-Gly external aldimine is bound inside the active site of *Mt*SHMT3 in a manner very similar to that of PLP-Ser (**Figure 8** and Supplementary Figure S2G). The hydrogen-bonding network is preserved despite lack of the hydroxymethyl group. At this point, we must also note that owing to the data resolution we cannot unambiguously determine whether the complex represents the PLP-Gly external aldimine, PLP-stabilized carbanion, quinonoid or an average of the three states. Furthermore, the PLP-Gly external aldimine may be a result of the forward (Ser degradation) or the reverse reaction (Ser synthesis) initiated by Gly binding to PLP. Nonetheless, comparison of the PLP-Ser and PLP-Gly complexes suggests that the formaldehyde release is not accompanied by conformational changes of the protein.

In the last step of the Ser-to-Gly biotransformation, regardless whether H_4_PteGlu_n_-driven or not, Gly is freed through imine exchange by Nζ amine of the PLP-binding Lys residue (Schirch and Szebenyi, 2005). Comparison of complexes with PLP-Gly external aldimine (**Figures 8A,B**) versus that with free Gly and K318-PLP internal aldimine (**Figures 8C,D** and Supplementary Figure S2H) revealed significant differences. As PLP-internal aldimine is restored, the pyridine ring rotates back by ∼20°; however, the hydrogen bond between the PLP O3 hydroxyl and Nδ of H292, present in the holo-*Mt*SHMT3, is not reestablished. H292, by its protonated N𝜀 interacts with one of the carboxyl oxygen atoms of the free Gly; the other O of Gly salt-bridges to R454. The amino group of Gly is H-bonded to Oη of Y144^∗^ and carboxyl of E136^∗^. Y144^∗^ changes the conformation dramatically, with a rotation of the phenyl ring by ∼90°, which restores the conformation observed in the holo-*Mt*SHMT3.

**FIGURE 9:**
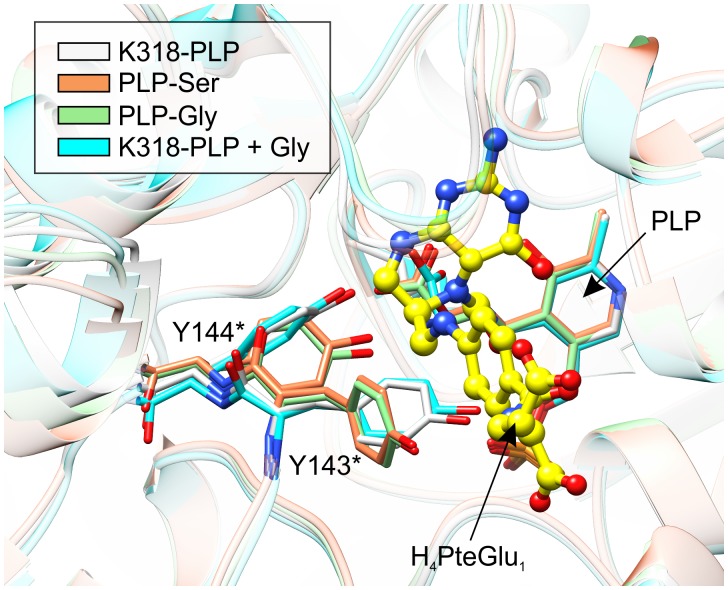
Comparison of the active-site architecture in the four snapshots along the course of the reaction. Yellow ball-and-stick model shows H_4_PteGlu_1_, superposed from mcSHMT complex (PDB ID: 1eji; Szebenyi et al., 2000). Note the concerted conformational changes of Y143^∗^ and Y144^∗^. The structures are colored according to the legend in the upper-left corner.

When *Mt*SHMT3 complexes representing four stages of the enzymatic reaction are compared, it becomes clear that the most spectacular variations concern Y143^∗^ and Y144^∗^ (**Figure 9**). It is also very interesting to see that movement of the two Tyr residues is apparently concerted. Above, we proposed that Y144^∗^, activated by the PLP-Ser carboxyl group, may be the base that abstracts the γ-hydroxyl proton of PLP-Ser in the H_4_PteGlu_n_-independent reaction. The role of Y144^∗^ in the H_4_PteGlu_n_-dependent reaction is still unclear, unlike the function of Y143^∗^ whose corresponding residue in *Bs*SHMT (Y60^∗^) has been shown to stack *p*-aminobenzoic acid moiety (PABA) of H_4_PteGlu_n_ (Pai et al., 2009). In this view, the conformational changes of Y143^∗^ in *Mt*SHMT3 are even more exciting. Superposition with mcSHMT in complex with H_4_PteGlu_1_ (PDB ID: 1eji; Szebenyi et al., 2000) revealed that Y143^∗^ of *Mt*SHMT3 is at a position able to accept PABA only in the PLP-Ser and PLP-Gly external aldimine complexes, that is, stages of the reaction in which H_4_PteGlu_n_ is desired to bind. Contrastingly, in the holo-structure, and in the complex with free Gly and internal aldimine (K318-PLP) Y143^∗^ would create a steric hindrance, preventing the binding of H_4_PteGlu_n_ (**Figure 9**). It is thus very likely that such a concerted movement of Y143^∗^ and Y144^∗^ may govern the enzyme inhibition by various folates, shown for many SHMTs (Matthews et al., 1982; Stover and Schirch, 1991; Zhang et al., 2010). It may also explain why we were not able to obtain a complex with H_4_PteGlu_1_ by soaking or cocrystallization – the gate-keeping Y143^∗^ prevented the folate from binding. Unfortunately, when the Ser-soaked crystals were also soaked with H_4_PteGlu_1_, resolution of the obtained diffraction data (below 3.5 Å) did not allow to model H_4_PteGlu_1_.

## Conclusion and Future Outlook

SHMT enzymes have been studied for a long time because they are promising targets for the design of antitumor, antibiotic and herbicide agents. Based on the phylogenetic analysis and prediction of the subcellular localization, plant cells contain SHMTs in the cytosol, mitochondria, chloroplasts, and nuclei. The presented herein structures of chloroplastic *Mt*SHMT3, which forms a tetramer, bring new insights into the complex metabolism of Ser and one-carbon units. As a member of the α-class of PLP enzymes, *Mt*SHMT3 binds the PLP prosthetic group in the center of the large domain. Binding of PLP is accompanied by a local disorder-to-order transition but does not involve large rearrangements, such as those observed in hmSMHT2 (Giardina et al., 2015). Soaking the *Mt*SHMT3 crystals with Ser in the absence of H_4_PteGlu_n_ allowed to capture intermediate states of the H_4_PteGlu_n_-independent reaction, proving at the same time that the enzyme is active *in crystallo*. The complex with PLP-Ser external aldimine shows its unique conformation, with the PLP-Ser γ-hydroxyl group hydrogen-bonded to Y144^∗^ and synperiplanar to the PLP-Ser carboxyl C atom. This lineup, whereby the hydroxyl of Y144^∗^ also interacts with the PLP-Ser carboxyl, suggests Y144^∗^ as a potential base in the H_4_PteGlu_n_-independent retroaldol cleavage. However, this hypothesis needs to be verified by a thorough functional study because different roles have been assigned to the equivalent Tyr residues of other SHMT enzymes (Contestabile et al., 2000; Rao et al., 2000). Unfortunately, a simple site-directed mutagenesis might bring biased results because Y144^∗^ is likely activated by the carboxyl group of the PLP-Ser intermediate.

Another novel feature observed on the basis of the *Mt*SHMT3 structures is the collaborative movement of Y143^∗^ and Y144^∗^. Y143^∗^ is shown to adopt a conformation ready to stack H_4_PteGlu_n_ in the states wherein the cosubstrate is needed in the active site (PLP-Ser and PLP-Gly external aldimines). On the other hand, in the PLP internal aldimine complexes (with and without free Gly) Y143^∗^ is rotated by ∼90° and would likely prevent H_4_PteGlu_n_ binding. We propose that the coordinated shift of the two Tyr residues is considered during the design of novel drugs. Moreover, since the plant SHMTs show more similarity with the mammalian than with the bacterial homologs, it is possible that the results obtained within the scope of this work may be relevant to the human enzymes as well. It is our hope that, with the current structural data, both efficiency and specificity of SHMT-targeted agents improve.

## Accession Numbers

PDB codes: 6ccz, *Mt*SHMT3 soaked with selenourea; 6cd0, *Mt*SHMT3 in holo- and apo- forms; 6cd1, *Mt*SHMT3 in complexes with the reaction intermediates.

## Author Contributions

MR and BS designed and performed the studies. MR analyzed the results and wrote the manuscript. AR performed and described the ConSurf analysis. ZD analyzed the results and supervised the work.

## Conflict of Interest Statement

The authors declare that the research was conducted in the absence of any commercial or financial relationships that could be construed as a potential conflict of interest.
