# Supplementary material for: Chloroplastic Serine Hydroxymethyltransferase From Medicago truncatula: A Structural Characterization
Source: Front Plant Sci. 2018 May 11;9:584. doi: 10.3389/fpls.2018.00584 (PMC5958214; doi:10.3389/fpls.2018.00584)
Supplement: Supplementary file 1 [file Image_1.PDF]

Supplementary Material

Chloroplastic serine hydroxymethyltransferase from *Medicago truncatula*: a structural characterization

Milosz Ruszkowski[1]*, Bartosz Sekula[1], Agnieszka Ruszkowska[2] and Zbigniew Dauter[1]
[1] Synchrotron Radiation Research Section of MCL, National Cancer Institute, Argonne, IL, USA
[2] Department of Chemistry and Biochemistry, University of Notre Dame, Notre Dame, IN, USA

[*]**Correspondence:** Milosz Ruszkowski: milosz.ruszkowski@nih.gov

Supplementary Figures:

Figure S1. Sequence alignment of seven SHMT sequences from *Medicago truncatula*.

Figure S2. Additional electron density maps.

Figure S3. Superposition of *Mt*SHMT3 structures from this work

Figure S4. Size exclusion profile of *Mt*SHMT3.

```
                        60        70                             80        90        100       110       120
                    ....|....|....|....|....                      |....|....|....|....|....|....|....|....|...
MtSHMT1     26  --------VYYKSSLPDEAVYDKENSRVSW----------------PKQLNSSLEEIDPEIADIIELEKARQWKGLELIPSENFTSLSVM 92
MtSHMT2     22  ------------SSLSAQHTH---KSHPDW----------------IKQLNDPLGVVDPEIEDIIELEKARQWKGLELIPSENFTSLSVM 81
MtSHMT3     55  ------ATFGKSSSSSSSPSSLPKKIGGDG----------------SSFLDYGLSEADPDVHAIINKEKDRQFRSLELIASENFTSKAVM 123
MtSHMT4     1   ---------------------------MDP---------------VSEWGNTPLVTVDPEIHDLIEKEKRRQCRGIELIASENFTSFAVI 48
MtSHMT5     1   ---------------------------MDP---------------VSEWGNTPLVTVDPEIHDLIEKEKRRQCRGIELIASENFTSFAVI 48
MtSHMT6     85  ILGHSMCIKRRRDSES--STITTKRASVESDRGDRLLDLESRKAAVRSWGDQPLQVADPDIYEIIEKEKKRQFKGIELIASENFVCRAVM 172
MtSHMT7     84  FMGRPICFKRRLEEETQQSFVPAKRVAVDS--------------KAVSWGNQPLSVADPEIFTIMEKEKLRQFKGIELIASENFVCRAVM 159

                      130       140       150       160       170       180       190       200       210
                .|....|....|....|....|....|....|....|....|....|....|....|....|....|....|....|....|....|...
MtSHMT1     93  QAVGSIMTNKYSEGYPGARYYGGNEYIDMAETLCQKRALEAFRLDPAKWGVNVQPLSGSPSNFHVYTALLKPHDRIMALDLPHGGHLSHG 182
MtSHMT2     82  QAVGSVMTNKYSEGYPGARYYGGNEYIDMAETLCQKRALETFGLDPTQWGVNVQSLSGSPSNFQVYTALLKPHERIMALDLPHGGHLSHG 171
MtSHMT3     124 EAVGSCLTNKYSEGLPGKRYYGGNEHIDELEILCQQRALAAFHLDGDKWGVNVQPLSGSPANFAVYTAILKPHDRIMGLDLPHGGHLSHG 213
MtSHMT4     49  EALGSALTNKYSEGMPGNRYYGGNEFIDQIENICRSRALTAFHLDAATWGVNVQPYSGSPANFAAYTAVLNPHDRIMGLDLPSGGHLTHG 138
MtSHMT5     49  EALGSALTNKYSEGMPGNRYYGGNEFIDQIENLCRSRALQAFHIDPQSWGVNVQPYSGSPANFAAYTAVLNPHDRIMGLDLPSGGHLTHG 138
MtSHMT6     173 EALGSHLTNKYSEGMPGARYYGGNQYIDEIETLCCERALAAFNLDPKCWGVNVQPYSCTSANFAVYTGLLAPGDRIMGLDTPSGGNTSHG 262
MtSHMT7     160 EALGSHLTNKYSEGMPGAKYYTGNQYIDQLEFLCCERALNAFHLDSSNWGVNVQPYSCTSANFAVYTGLLNPGDRIMGLDSASGGHLSHG 249

                       220       230       240       250       260       270       280       290       300
                .|... .|....|....|....|....|....|....|....|....|....|....|....|....|....|....|....|....|..
MtSHMT1     183 YQTDT-KKISAVSIFFETMPYRLDESTGYIDYDQLEKSATLFRPKLIVAGASAYARLYDYARIRKVCDKQKAVMLADMAHISGLVAAGVI 271
MtSHMT2     172 YQTDT-KKISAVSIFFETMPYRLDESTGYIDYDQMEKSAALFRPKLIVAGASAYARLYDYARIRKVCDKQKAVMLADMAHISGLVAAGVI 260
MtSHMT3     214 FMTAK-RRVSGTSIYFESMPYRLDESTGVIDYDMLEKTAALFRPKLIIAGASAYPRDIDYARFRKIADSVGAFLMMDMAHVSGLIAASVL 302
MtSHMT4     139 YYTSGGKKISATSIYFESLPYKVNSTTGFIDYDRLEEKALDFRPKLIICGGSAYPRDWDYGRFRQVADKCGALLLCDMAHISGLVAAQEA 228
MtSHMT5     139 YYTSGGKKISATSIYFESLPYKVNSTTGFIDYDRLEEKALDFRPRLIICGGSAYPRDWDYKRFRDVADKCGALLLCDMAHFSGLVAAQEV 228
MtSHMT6     263 YYTPNGKKVSGASIFFESLAYKINPQSGFIDYDKLEERALDFRPKILICGGSSYPREWDYARFRHVADKCGAVLLCDMAQISGIIAAKEC 352
MtSHMT7     250 YYTHGGKKVSAASIFFETLPYKVNPLTGYIDYDKVEEKAVDYRPKILICGGSSYPREWDYARFRKIADKCGAVLMCDMAHISGLVAAREV 339

                       310       320       330                     340       350       360       370
                ..|....|....|....|....|....|..   ..|..          ..|....|....|....|....|....|....|....|...
MtSHMT1     272 PSPFDYADVVTTTTHKSLRGPRGAMIFFRKGLKEVNKQ------GKEVFYDYEDKINQAVFPGLQGGPHNHTITGLAVALKQATTPEYKA 355
MtSHMT2     261 PSPFDYADVVTTTTHKSLRGPRGAMIFFRKGLKEINKK------GQEVLYDYEDKINQAVFPGLQGGPHNHTITGLAVALKQAMTPEFKN 344
MtSHMT3     303 ADPFEFVDIVTTTTHKSLRGPRGGMIFFKK--DAVHG------------VDLESAINNAVFPGLQGGPHNHTIGGLAVCLKYAQSPDFKN 378
MtSHMT4     229 NDPFAFCDIVTTTTHKSLRGPRAGMIFYRKGPKPPKKG-----QPENAVYDFEDKINFAVFPSLQGGPHNHQIGALAVALKQATTPGFKA 313
MtSHMT5     229 NNPFEYCDIVTTTTHKSLRGPRAGMIFYRKGPKPPKKG-----QPENAVYDFEDKINFAVFPSLQGGPHNHQIGALAVALKQAMSPGFKA 313
MtSHMT6     353 VNPFDYCDVVTSTTHKSLRGPRGGIIFYRKGTKPRKRGILLTQGHESDQYDFEEKINFAVFPSLQGGPHNNHIAALAIALKQVATPEYKA 442
MtSHMT7     340 ASPFDYCDIVTSTTHKSLRGPRGGIVFYRKGPKPRKQGFVLNHGDDNSNYDFEEKINFALYPSLQGGPHNNHIAALAIALKQVATPEYKA 429

                380       390       400       410       420       430       440       450       460
                .|....|....|....|....|....|....|....|....|....|....|....|....|....|....|....|....|....|...
MtSHMT1     356 YQEQVLSNCAKFAQALSEKGYELVSGGTENHLVLVNLKNKGIDGSRVEKVLEAVHIAANKNTVPGDVSAMVPGGIRMGTPALTSRGFVEE 445
MtSHMT2     345 YQKQVLSNSSTFAQSLLEKGYDLVSGGTENHLVLVNLRNKGIDGSRVEKVLESVHIAANKNTVPGDVSAMVPGGIRMGTPALTSRGFVED 434
MtSHMT3     379 YQNQVVANCRALANRLVEHEYKLVSGGSDNHLVLVDLRPSGIDGARVEKILDMASITLNKNSVPGDKSALVPGGIRIGSPAMTTRGLGEK 468
MtSHMT4     314 YAKQVKANAVALGNYLISKGYSLVTGGTENHLVLWDLRPLGLTGNKVEKLCDLCNITVNKNAVFGDSSALAPGGVRIGAPAMTSRGLVEK 403
MtSHMT5     314 YAKQVKANAVAIGNYLMSKGYSLVTGGTENHLVLWDLRPLGLTGNKVEKLCDLCNITVNKNAVFGDSSALAPGGVRVGAPAMTSRGLVEK 403
MtSHMT6     443 YMQQVKKNAQALASALLRRKCRLVTGGTDNHLILWDLRPLGLTGKFYEKVCEACHITLNKIAIFGDNGIIIPGGVRIGTPAMTSRGCLEA 532
MtSHMT7     430 YMQQVKRNAQALATALLKRKCRLVTDGTDNHLLLWDITALGLIDRNYEKVCEACHITLNKCAIYGS---ISSGGVRIGTPAMTSRGCLED 516

                470       480       490       500       510       520       530
                .|....|....|....|....|....|....|....|....|....|....|....|....|...
MtSHMT1     446 DFVKVAEYFDASVNLALKIKAESKGTKLKDFVETLQSSSY-VQSEISKLRHDVEEFAKQFPTIGFEKSSMKYNK 518
MtSHMT2     435 DFKKVAEYFDAAVKIALQIKENSKGTKLKDFVEAMESDSQ-VQSQIADLRHDVEGYAKQFPTIGFEIETMKYNK 507
MtSHMT3     469 EFELIADLIHEGVRISLEAKSLVSGTKVQDFLNFVLAPEFPLGDKVSNLRRKVEALATQYPIPGV--------- 533
MtSHMT4     404 DFEQIAEFLHRAVTLTLEIQK-EYGKLLKDFNKGLVNNKA-----LEDLKADVEKFSASFDMPGFLVSELKYKD 471
MtSHMT5     404 DFEKIGEFLHRAVTLTLEIQK-EHGKLLKDFNKGLVDNKA-----IAELKADVEKFSSLFGMPGFLVSEMKYKD 471
MtSHMT6     533 DFETMADFLFRAAQIANMLQR-EHGKLQKTISKVLESNRD-----ILELRARVEAFATQFALPGFDI------- 593
MtSHMT7     517 DFETMADFLLRAAQITSIIQR-EHGKSCKDFLKGLQSNKD-----IFDLRNRVETFGSQFEMPGFNY------- 577
```

Supplementary figure S1

Sequence alignment of seven SHMT sequences from *Medicago truncatula*. The sequence of *Mt*SHMT3, is in bold. UniProt accession numbers [in square brackets] are as follows: MtSHMT1 [A9YWS0], MtSHMT2 [G7K5Z4], MtSHMT3 [G7ILW0], MtSHMT4 [G7JAR7], MtSHMT5 [G7LH57], MtSHMT6 [G7IPE1], and MtSHMT7 [G7J467].

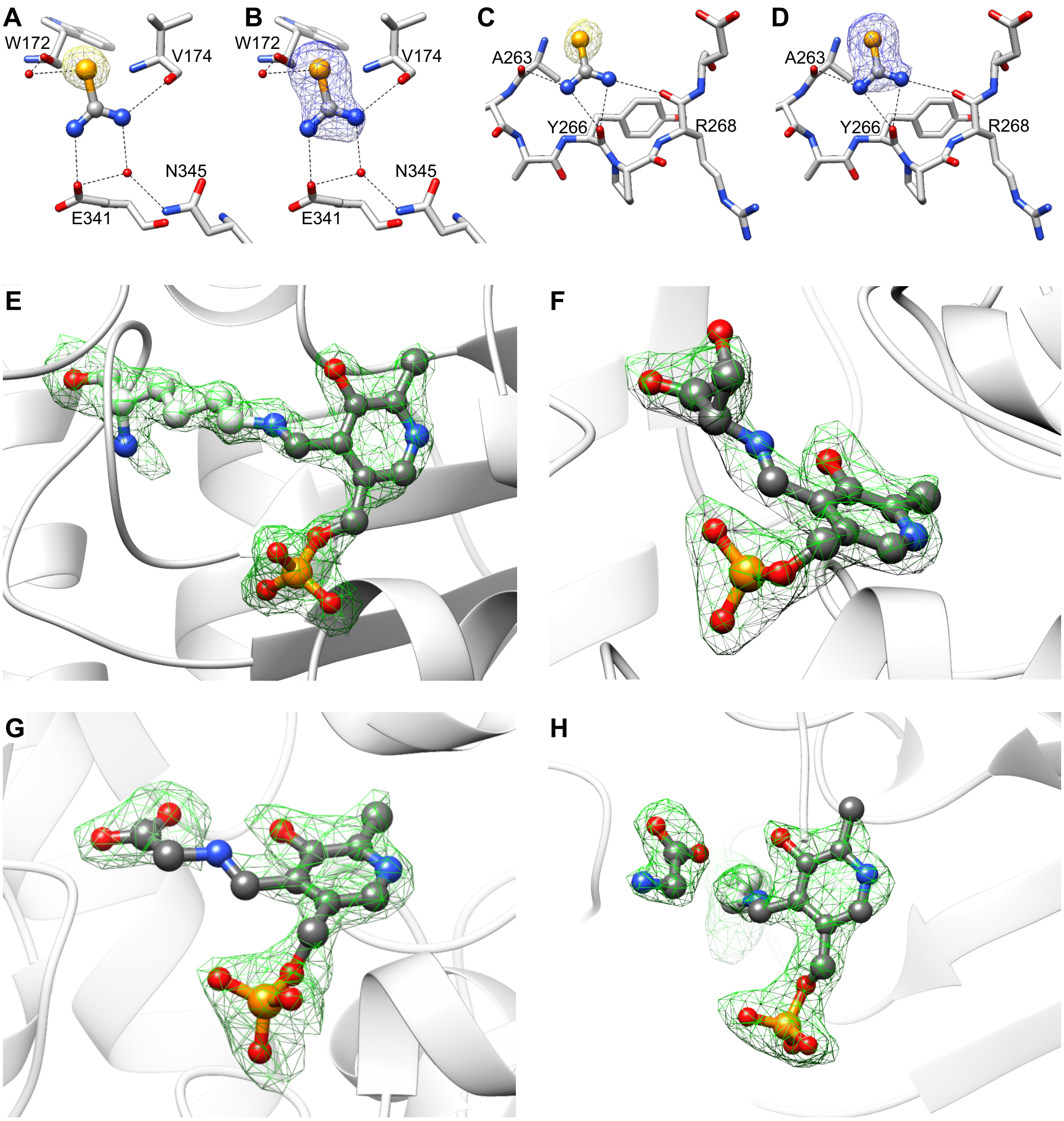

Supplementary figure S2

Additional electron density maps. Panels A-D show two examples of the selenourea binding sites. Yellow mesh represents anomalous difference map contoured at 15 σ level, whereas blue mesh is the $2F_o$-F_c map (1.5 σ). Panels F-H show OMIT F_o-F_c electron density maps (green mesh) for: (E) PLP internal aldimine, (F) PLP-Ser external aldimine, (G) PLP-Gly external aldimine, and (H) PLP internal aldimine with free Gly. In panel E, the map level is 4 σ, whereas in F-H the level is 3 σ.

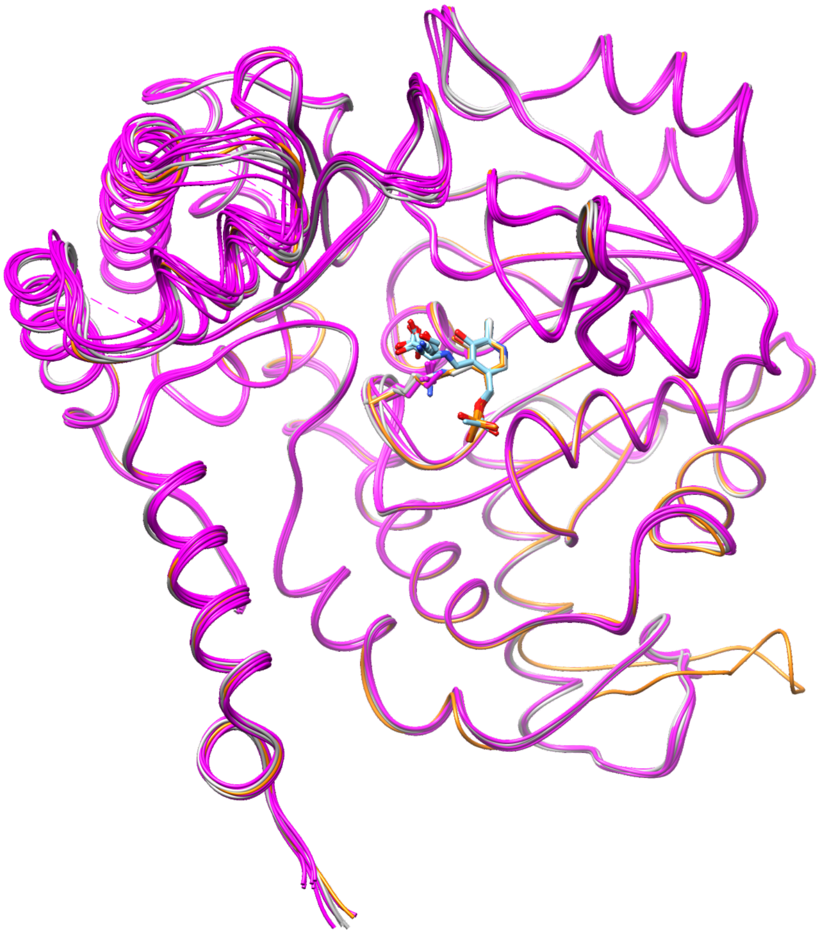

Supplementary figure S3
Comparison of the twelve protein subunits showing *Mt*SHMT3 in apo-state (orange), holo-state (gray), and with reaction intermediates (magenta).

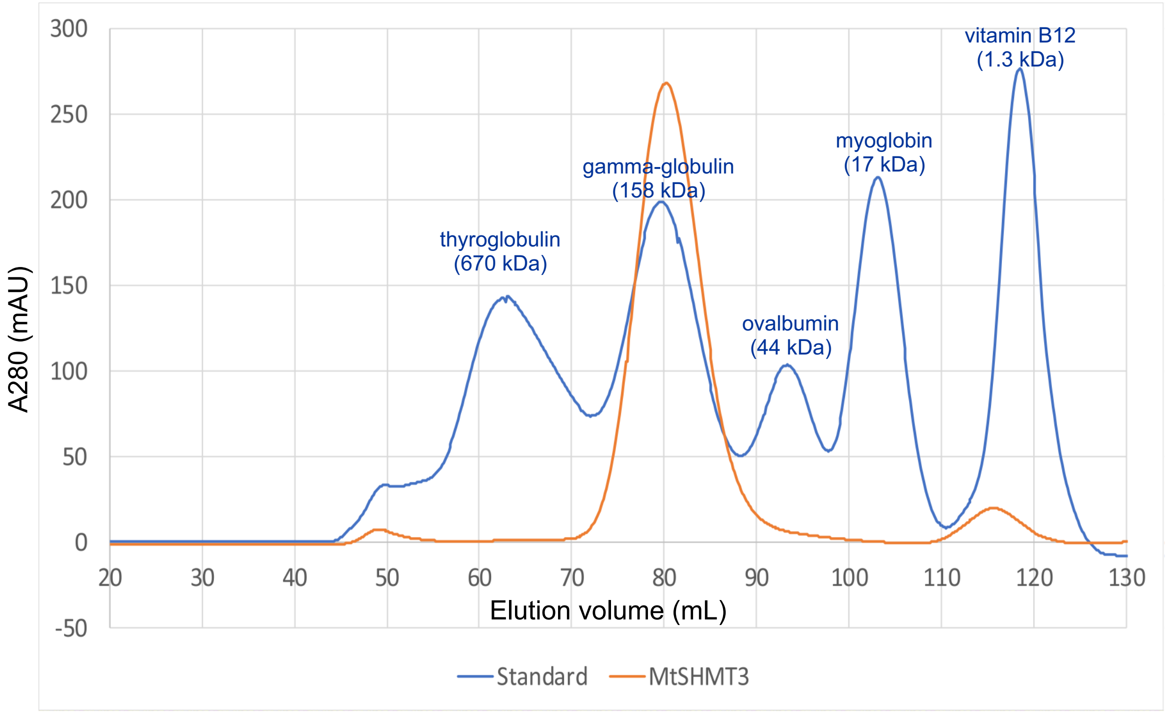

Supplementary figure S4

Size exclusion profile of *Mt*SHMT3 against standard globular proteins.
